# Arthritis Induces Early Bone High Turnover, Structural Degradation and Mechanical Weakness

**DOI:** 10.1371/journal.pone.0117100

**Published:** 2015-01-24

**Authors:** Bruno Vidal, Rita Cascão, Ana Catarina Vale, Inês Cavaleiro, Maria Fátima Vaz, José Américo Almeida Brito, Helena Canhão, João Eurico Fonseca

**Affiliations:** 1 Instituto de Medicina Molecular, Faculdade de Medicina da Universidade de Lisboa, Lisbon, Portugal; 2 Instituto de Ciência e Engenharia de Materiais e Superfícies, Instituto Superior Técnico, University of Lisbon, Lisbon, Portugal; 3 Instituto Superior de Ciências da Saúde Egas Moniz—Campus Universitário, Quinta da Granja, Caparica, Portugal; 4 Departamento de Engenharia Mecânica, Instituto Superior Técnico, UL, Lisbon, Portugal; 5 Rheumatology Department, Lisbon Academic Medical Centre, Lisbon, Portugal; University of East London, UNITED KINGDOM

## Abstract

**Background:**

We have previously found in the chronic SKG mouse model of arthritis that long standing (5 and 8 months) inflammation directly leads to high collagen bone turnover, disorganization of the collagen network, disturbed bone microstructure and degradation of bone biomechanical properties. The main goal of the present work was to study the effects of the first days of the inflammatory process on the microarchitecture and mechanical properties of bone.

**Methods:**

Twenty eight Wistar adjuvant-induced arthritis (AIA) rats were monitored during 22 days after disease induction for the inflammatory score, ankle perimeter and body weight. Healthy non-arthritic rats were used as controls for compar-ison. After 22 days of disease progression rats were sacrificed and bone samples were collected for histomorphometrical, energy dispersive X-ray spectroscopical analysis and 3-point bending. Blood samples were also collected for bone turnover markers.

**Results:**

AIA rats had an increased bone turnover (as inferred from increased P1NP and CTX1, *p* = 0.0010 and p = 0.0002, respectively) and this was paralleled by a decreased mineral content (calcium *p* = 0.0046 and phos-phorus *p* = 0.0046). Histomorphometry showed a lower trabecular thickness (*p* = 0.0002) and bone volume (*p* = 0.0003) and higher trabecular sepa-ration (*p* = 0.0009) in the arthritic group as compared with controls. In addition, bone mechanical tests showed evidence of fragility as depicted by diminished values of yield stress and ultimate fracture point (*p* = 0.0061 and *p* = 0.0279, re-spectively) in the arthritic group.

**Conclusions:**

We have shown in an AIA rat model that arthritis induc-es early bone high turnover, structural degradation, mineral loss and mechanical weak-ness.

## Introduction

Rheumatoid arthritis (RA) is a chronic immune-mediated inflammatory disease, which affects around 1% of the world-population[1]. It causes joint and systemic inflammation that is reflected in local and systemic bone damage[2]. Bone is a dynamic tissue composed mainly of a type I collagen matrix that constitutes the scaffold for calcium hydroxyapatite crystal deposition. Remodelling of bone is a continuous process by which osteoclasts resorb bone tissue and osteoblasts produce new bone matrix that is subsequently mineralised. Biochemical markers of this bone turnover are produced and released into circulation, providing a read-out of kinetics and the balance between bone loss and formation. More specifically, bone-resorbing osteoclasts release carboxy-terminal collagen cross-linking telopeptides (CTX-I), a marker for bone degradation, which is produced by cathepsin K that is involved in systemic bone resorption [3]. During bone formation, collagen is synthesized by osteoblasts in the form of procollagen. This precursor contains a short signal sequence and terminal extension peptides: amino-terminal propeptide (PINP) and carboxy-terminal propeptide. These propeptide extensions are removed by specific proteinases before the collagen molecules form. PINP can be found in the circulation and its concentration reflects the synthesis rate of collagen type I, being thus a marker of bone formation [4]. As RA progresses there is marked articular destruction and decreased joint mobility with radiological evidence of erosion with significant impact on life quality within 2 years of disease onset [5]. In addition, osteoporosis is a common finding in patients with RA [6] and is responsible for increased rates of vertebral and hip fractures in these patients [7, 8]. RA is associated with an increased expression of the receptor activator of RANKL (receptor activator of nuclear factor kappa–B ligand, NF-KB ligand) and low levels of its antagonist, osteoprotegerin (OPG) [9]. In addition, very early on in the disease process, RA serum and synovial fluid present a cytokine profile, including interleukin (IL) 1, IL6, IL17 and tumour necrosis factor (TNF), which further favours osteoclast differentiation and activation[10–12]. Evidence suggests that bone remodelling disturbances in RA contribute not only to local bone erosions but also to the development of systemic osteoporosis [13].

We have previously found in a chronic animal model of arthritis (SKG mouse model) that prolonged inflammation (5 and 8 months) directly leads to the degradation of bone biomechanical properties, namely stiffness, ductility and bone strength, which was paralleled by a high collagen bone turnover and disorganization[4, 12, 14, 15]. Based on the fact that most of the effectors of bone metabolism are engaged in the disease process since the early phase, we now hypothesise that this process starts upon the first inflammatory manifestations[10–12]. To test this we selected the adjuvant-induced arthritis (AIA) model in rats, characterized by a rapid onset polyarticular inflammation and widely used for testing new treatments for arthritis [16–18]. Understanding the systemic inflammatory consequences on bone would expand the use of this model also for testing new drugs with potential bone therapeutic effects.

The main goal of the present work was to study, in a rat model of AIA, the effects of the first days of the systemic inflammatory process on the microarchitecture and mechanical properties of bone.

## Materials and Methods

### Animal experimental design

Twenty-eight Wistar AIA rats were purchased from Charles River Laboratories International (Massachusetts, USA). Eight-week-old females weighing 200–230 g were maintained under specific pathogen free (SPF) conditions. All experiments were approved by the Animal User and Ethical Committees of the Instituto de Medicina Molecular, Lisbon University, according to the Portuguese law and the European recommendations. Animals were sacrificed when presenting an inflammatory score (0–3) of 3 in 2 paws or when presenting 20% of body weight loss.

Rats were housed per groups (healthy *vs* arthritic) under standard laboratory conditions (at 22°C under 12-hour light/12-hour dark conditions). The inflammatory score, ankle perimeter and body weight were measured during the study period. Inflammatory signs were evaluated by counting the score of each joint in a scale of 0–3 (0— absence; 1— erythema; 2— erythema and swelling; 3— deformities and functional impairment). The total score of each animal was defined as the sum of the partial scores of each affected joint [19]. Rats were sacrificed by CO^2^narcosis after 22 days of disease evolution and blood as well as bone samples were collected.

### Bone remodelling markers quantification

Serum samples were collected at the time of sacrifice and stored at -80°C. Bone remodelling markers CTX I (C-terminal telopeptides of type-I collagen) and P1NP (total procollagen type 1 N-terminal propeptide) were quantified by Serum Rat-Laps ELISA assay (Immunodiagnostic Systems Ltd, Boldon, UK), according to the provider’s instructions.

### Bone histomorphometry

The 4^th^ lumbar vertebrae (L4) were collected from each animal at sacrifice for histomorphometric analysis. Samples were fixed immediately in ethanol 70% and then dehydrated with increasing ethanol concentrations (96% and 100%). Samples were next embedded in methylmetacrylate (MMA) solution. Serial transversal sections through L4 were performed with 5-μm-thick and stained with Aniline Blue in order to distinguish bone and bone marrow, allowing bone structural analysis. Images were acquired using a Leica DM2500 microscope equipped with a colour camera Leica CCD Camera (Leica microsystems, Wetzlar, Germany)[20].

All variables were expressed and calculated according to the recommendations of the American Society for Bone and Mineral Research [21], using a morphometric program (Image J 1.46R with plugin Bone J).

Ratio of trabecular bone volume / total tissue volume, trabecular thickness and trabecular separation were evaluated by standard histomorphometric parameters at x12.5 magnification.

### Energy dispersive X-ray spectroscopy analysis

Energy dispersive X-ray spectroscopy is a sensitive qualitative and semi-quantitative technique to evaluate the mineral content in bone. The quantitative information is based on the relative elemental abundance.

Using a standard system, semi-quantitative X-ray fluorescence measurements were performed in cortical and trabecular bone powder samples, with the purpose of quantifying calcium and phosphorus concentration.

After excision, fresh femurs were freeze dried for 46 hours, with a multipurpose ice condenser (ModulyoD-230, Thermo Savant, Schwerte, Germany) operated at a nominal temperature of -50°C, in order to remove excess of water.

The semi-quantitative measurements of bone powder were performed with a 4 kW commercial wavelength dispersive X-ray fluorescence spectrometer (Bruker S4 Pioneer, Karlsruhe, Germany), using a Rh X-ray tube with a 75 mm Be end window and a 34 mm diameter collimator mask. Measurements were performed in helium mode and using high-density polyethylene X-ray fluorescence sample cups with 35.8 mm diameter assembled with a 4 mm prolene film to support the bone sample. The polyethylene cup was placed in steel sample cup holders with an opening diameter of 34 mm.

### Bone mechanical testing

Bone mechanical properties were evaluated by a three-point bending method using a electromechanical machine (model 5566, Instron Corporation, Canton, USA) using a load-cell of 500N. The femur was placed on a holding device with a support span distance of 5 mm (L), with the lesser trochanter proximal in contact with the proximal transverse bar. The load was applied at the mid-shaft of the diaphysis with a cross-head speed of 0.005 mm/s until the fracture occurred.

The stress-strain curve can be obtained from the load-displacement representation, with the initial dimensions of the sample, using engineering equations (S1B Fig.).

An example of a stress-strain curve obtained in the three point bending tests is shown in S1A Fig. The points of the yield stress and ultimate stress are indicated. This stress-strain curve can be broken down into pre-yield and post-yield portions. Pre-yield toughness represents the area under the stress/strain curve up to the yield point, which is where permanent deformation of the bone has occurred while post-yield toughness represents the area under the curve between the yield point and bone fracture. In these bending tests there is a significant amount of displacement between the yield point and the eventual fracture[22].

### Statistical analysis

Continuous variables were expressed by mean +- standard deviation (SD) or median and interquartile range. The normality distribution was assessed by D’Agostino and Pearson test. Statistical differences were determined with parametric *t*–test or non-parametric Mann Whitney test according variables distribution using GraphPad Prism (GraphPad, California, USA). Differences were considered statistically significant for *p* values ≤ 0.05.

## Results

### Inflammatory progress

**Figure 1:**
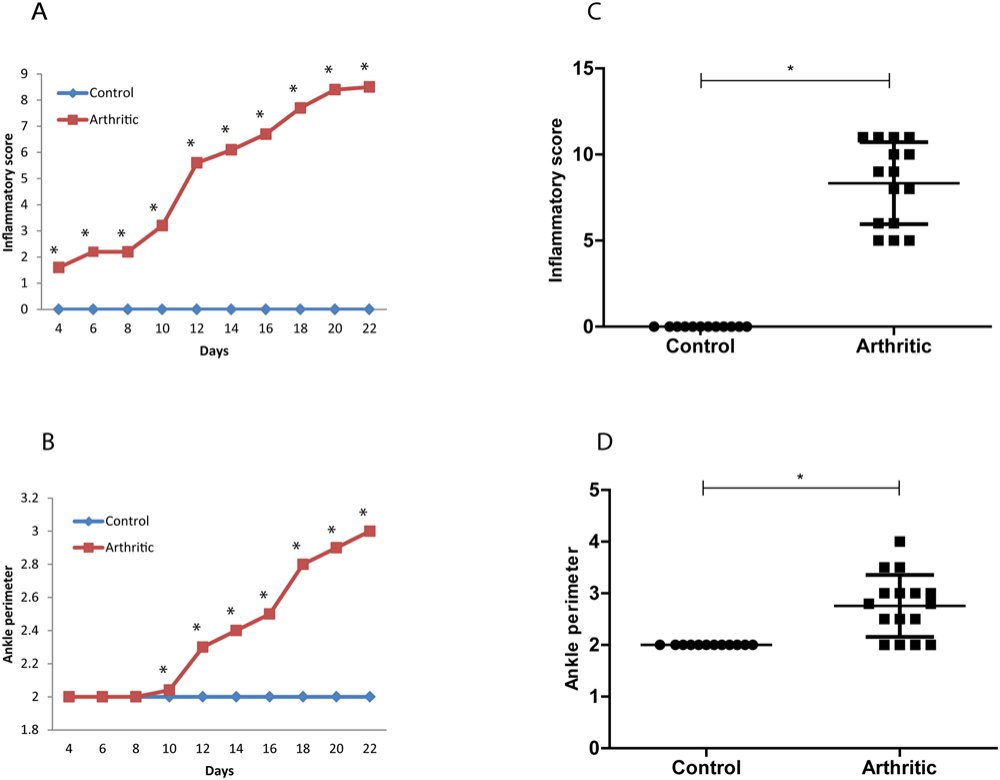
Inflammatory score (A) and ankle perimeter (B) throughout time. Inflammatory score (C) (p = 0.0037) and Ankle perimeter (D) (p = 0.0085) in control (N = 12) and arthritic groups (N = 16) by the time of sacrifice after 22 days post disease induction.

First, we validated the kinetic of disease development of the AIA rat model. Inflammatory signs (Fig. 1A) and ankle perimeter (Fig. 1B) were assessed throughout time, as shown in Fig. 1. All animals from the arthritic group (N = 16) presented arthritis signs by the fourth day post disease induction.

Statistical differences were determined with non-parametric Mann Whitney test using GraphPad Prism (GraphPad, California, USA). Differences were considered statistically significant for p values ≤ 0.05.

The initial acute inflammation was observed around day 4 and progressed during 22 days post disease induction. After 10 days of arthritis induction, the inflammatory manifestations increased sharply as depicted by an increase in ankle perimeter. Maximal swelling occurred at day 19 post disease induction. At day 22 post arthritis induction inflammatory score (Fig. 1C) and ankle perimeter (Fig. 1D) were significantly increased in the arthritic group (*p* = 0.0037 and *p* = 0.0085, respectively) in comparison with healthy control rats.

### Bone turnover markers

Bone resorption marker CTX I, which reflects osteoclastic activity, is a degradation product of type I collagen, the major structural protein of bone. While the bone formation marker P1NP, a bio product of type I collagen synthesis, is a marker for osteoblastic activity.

**Figure 2:**
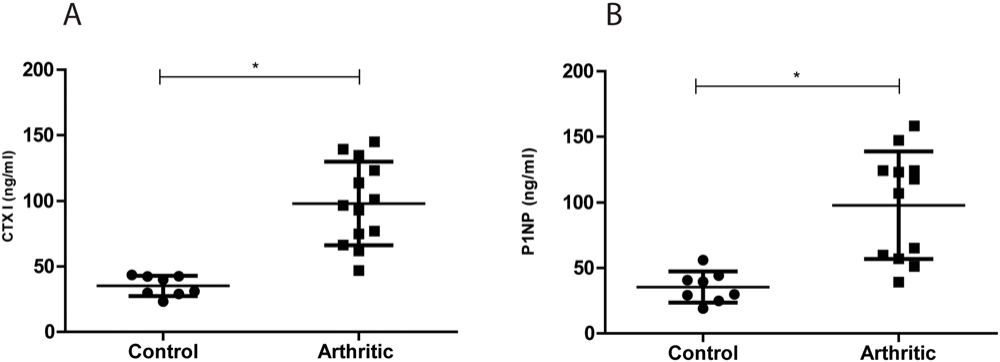
Bone turnover markers quantification in control (N = 9) and arthritic rats (N = 13). Serum samples collected at day 22 (sacrificed) were analysed by ELISA technique. Bone resorption marker, CTX I (A) and bone formation marker, P1NP (B) were increased in arthritic rats (p = 0.0002 and p = 0.0010, respectively).

We have observed that both CTXI (Fig. 2A) and P1NP (Fig. 2B) were significantly increased in the arthritic group in comparison with the healthy control animals (p = 0.0002 and p = 0.0010, respectively), revealing an increase of bone turnover in the arthritic group.

### Histomorphometry of bone

**Figure 3:**
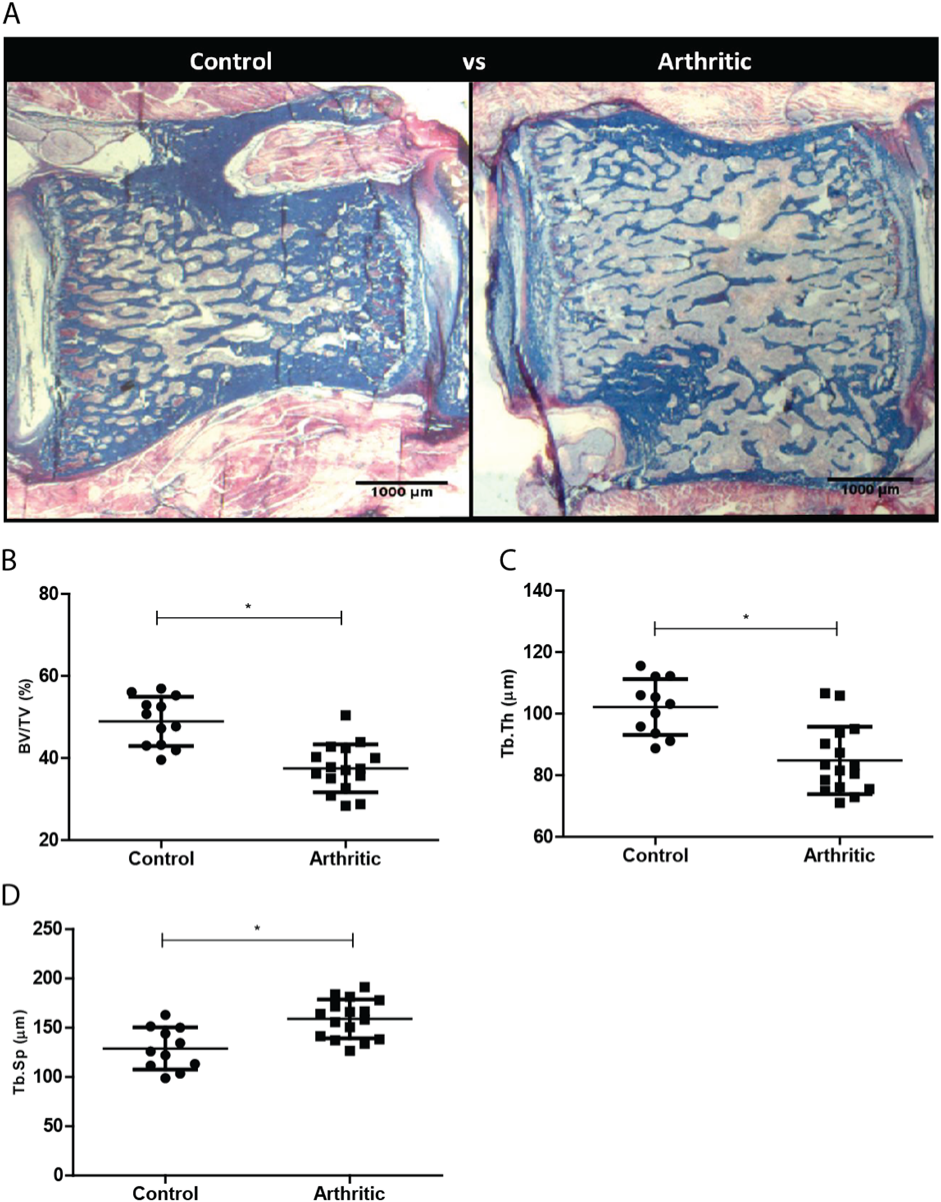
Bone histomorphometry assessment of the 4th lumbar vertebra (L4). Assessment of L4 in control (N = 12) and arthritic group (N = 16). (A) Illustrative Aniline blue stained sections of L4 vertebra collected at day 22 post disease induction (sacrifice). Bone volume per tissue volume or trabecular bone volume fraction (B) and trabecular thickness (C) were decreased in arthritic rats while trabecular separation (D) was increased. Magnification x12.5.

Bone histomorphometry was used to measure bone static parameters such as bone trabecular volume, trabecular thickness and trabecular separation in order to determine the effects of inflammation on bone microstructure (Fig. 3A).

Trabecular bone volume (*p* = 0.0003) (Fig. 3B) and trabecular thickness (*p* = 0.0002) (Fig. 3C) were significantly reduced in arthritic animals comparing with healthy control animals. Moreover, trabecular separation (*p* = 0.0009) (Fig. 3D) was significantly increased in the arthritic group, in comparison with healthy control rats.

### Energy dispersive X-ray spectroscopy

Calcium (Ca) and Phosphorus (P) are the most abundant elements present in bone mineral matrix. In fact, calcium has been reported as the most important nutrient associated with peak bone mass and may be the only one for which there is epidemiological evidence of a relation to fracture rate[23].

**Figure 4:**
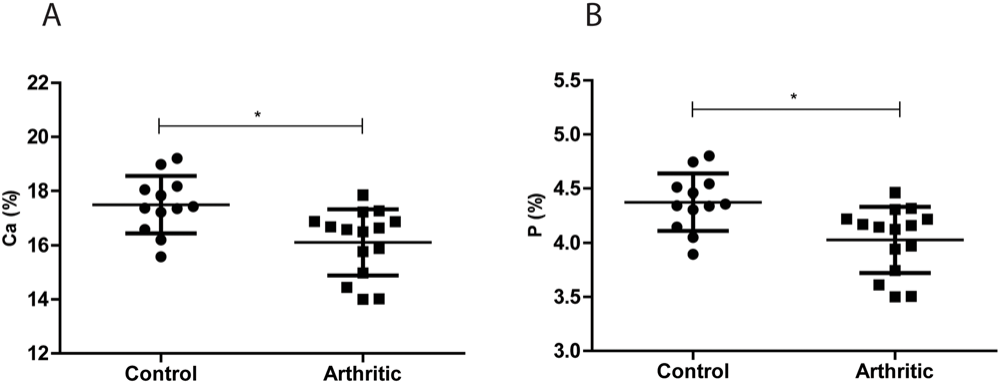
Calcium and Phosphorus bone content acquired by energy dispersive X-ray spectroscopy. Ca (A) and P (B) bone content were decreased in the arthritic group (N = 16) as compared with controls (N = 12). Bone powder was acquired from bone samples collected at day 22 post disease induction (sacrifice).

We used energy dispersive X-ray spectroscopy to quantify the calcium and phosphorus content in our samples. We have observed that Ca (*p* = 0.0046) (Fig. 4A) and P (*p* = 0.0046) (Fig. 4B) content were decreased in the arthritic group as compared to controls.

### Bone mechanics

**Figure 5:**
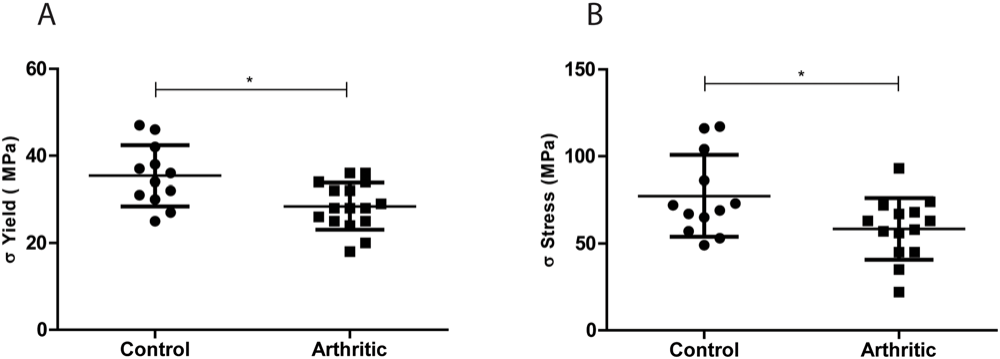
Mechanical analysis acquired by 3 point bending tests. Yield stress (A) and Ultimate stress (B) were decreased in arthritic rats (N = 16) as compared to controls (N = 12). Bone samples were collected at day 22 post disease induction (sacrifice).

The three-point-bending biomechanical tests aimed to explore the bone mechanical competence of both groups 22 days post disease induction. Results showed decreased values of yield stress (moment of occurrence of first micro fractures) (*p* = 0.0061) (Fig. 5A) and ultimate stress (moment of occurrence of complete fracture) (*p* = 0.0279) (Fig. 5B) in arthritic animals when compared to the control group.

## Discussion

In the present study, we demonstrated in an AIA rat model, that arthritis induces very early high bone turnover, trabecular degradation, mineral loss and mechanical weakness.

Biochemical markers of bone turnover were quantified in order to evaluate the impact of systemic inflammation on bone metabolism. An increased bone turnover activity was shown in arthritic animals, as depicted by increased CTXI and P1NP levels. This observation was consistent with previously published data showing the presence of a large number of osteoclasts in AIA bone [17]. Data already published by our group in another animal model of arthritis (the SKG mice model) have also shown that P1NP levels were increased in arthritic animals and so did CTX-I levels [4], reflecting an overall increase in bone turnover [24]. Studies on RA patients measuring P1NP have produced varying results, whereas measurements in CTX-I mostly show increased levels [25]. In RA patients bone metabolism is more active (increased P1NP) in earlier stages of the disease and a decrease in bone metabolic activity (both P1NP and CTX) occurs with disease progression, both showing correlation with tender and swollen joints [15]. Despite the existing variability, P1NP has been mainly found to be increased in RA patients when compared to controls, together with CTX-I, revealing a compensatory mechanism in bone turnover [26].

Due to increased bone turnover it was therefore of interest to assess the effects of inflammation on bone microstructure. Histomorphometric data revealed, in arthritic animals, a lower fraction of trabecular bone volume and a lower average trabecular thickness as well as a higher average trabecular separation, in comparison with controls. These findings were in line with the described bone volume loss, measured by uCT, in this rat model [17].

In addition, we quantified calcium and phosphorus content, the two major minerals present in bone [27], by energy dispersive X-ray spectroscopy. The arthritic group showed a significant decreased mineral content, when compared to the control group. This result corroborated an overall bone mineral loss, as a result of an unbalanced high bone turnover, which might lead to bone fragility and consequently fracture.

In accordance, mechanical tests revealed that arthritic femurs have a significantly lower yield stress and ultimate stress as compared to control femurs, meaning that bone is more fragile and prone to fracture.

In summary, we have shown, in an AIA rat model, that the systemic inflammation associated with a polyarthritis is able to induce an early high bone turnover, bone microarchitecture degradation, low mineral content and mechanical weakness. In addition, our results have expanded the knowledge on this model. In fact, our findings, suggest that AIA is a fast and adequate model to study the effects of arthritis on bone properties and consequently a potentially accurate model to study anti-arthritic compounds with bone protective effects.

## Supporting Information

S1 Fig Scheme representative of the yield stress and ultimate stress points in a stress/strain curve. Yield stress and ultimate stress points (A) obtained with bending test with the specific formulas for stress (B) strain (C) calculation, where σ—stress (Pa); L—load (N); s—support span (mm); df—femoral outer diameter (mm); ε—strain (%); Δl–displacement (mm).
(TIF)
