# Correction: Arthritis Induces Early Bone High Turnover, Structural Degradation and Mechanical Weakness

**DOI:** 10.1371/journal.pone.0137372

**Published:** 2015-09-16

**Authors:** Bruno Vidal, Rita Cascão, Ana Catarina Vale, Inês Cavaleiro, Maria Fátima Vaz, José Américo Almeida Brito, Helena Canhão, João Eurico Fonseca

The following information is missing from the Funding section: This study was supported by an unrestricted grant from UCB Pharma (www.ucb.com). The funder had no role in study design, data collection and analysis, decision to publish, or preparation of the manuscript.

The authors have also provided an updated Competing Interests statement: This study was supported by an unrestricted grant from UCB Pharma. João Eurico Cabral da Fonseca received this grant as the head of Rheumatology Research Unit at Instituto de Medicina Molecular, Faculdade de Medicina da Universidade de Lisboa. This does not alter our adherence to PLOS ONE policies on sharing data and materials.
