# Supplementary figures and images for: Arthritis Induces Early Bone High Turnover, Structural Degradation and Mechanical Weakness
Source: PLoS One. 2015 Jan 24;10(1):e0117100. doi: 10.1371/journal.pone.0117100 (PMC4305284; doi:10.1371/journal.pone.0117100)

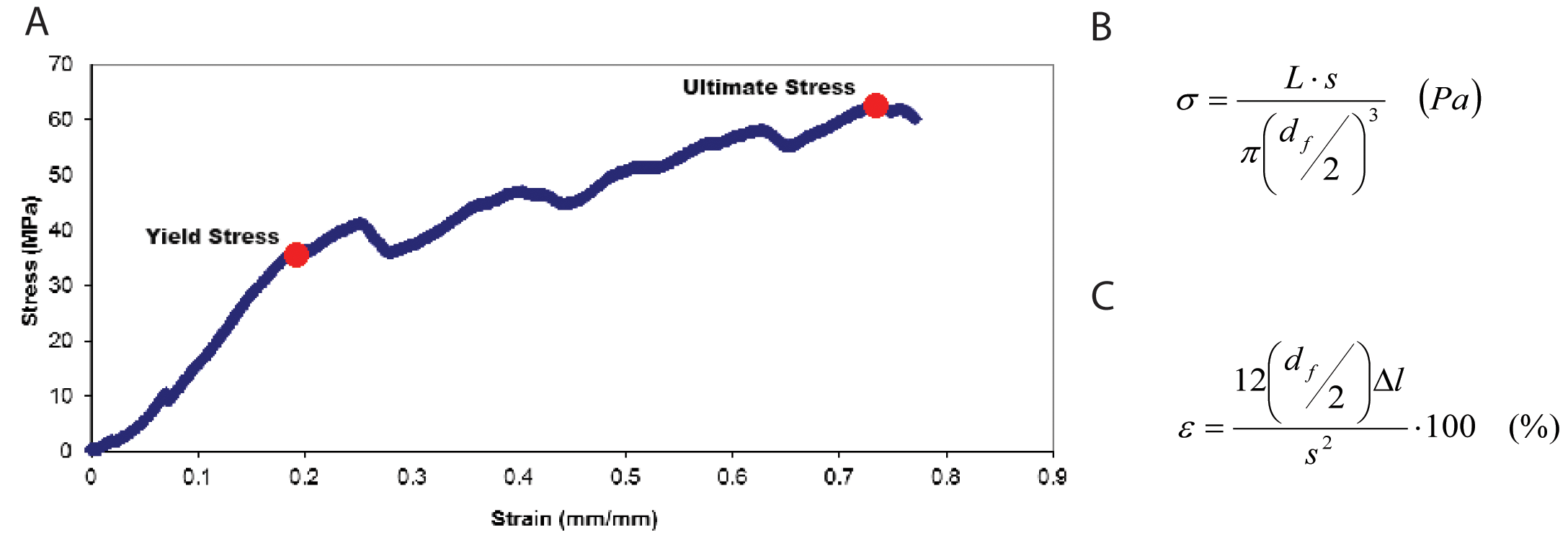

Supplement: S1 Fig — Yield stress and ultimate stress points (A) obtained with bending test with the specific formulas for stress (B) strain (C) calculation, where σ—stress (Pa); L—load (N); s—support span (mm); df—femoral outer diameter (mm); ε—strain (%); Δl–displacement (mm). (TIF) [file pone.0117100.s001.tif]
